# Proportion of carbapenem-resistant Enterobacterales with readily detectable beta-lactam resistance

**DOI:** 10.1017/ash.2025.10072

**Published:** 2025-08-07

**Authors:** Eli Wilber, Gillian Smith, Jesse T. Jacob, Paulina A. Rebolledo

**Affiliations:** 1 Division of Infectious Diseases, Department of Medicine, Emory University School of Medicine, Atlanta, GA, USA; 2 Grady Health System, Atlanta, GA, USA; 3 Georgia Emerging Infections Program, Atlanta, GA, USA; 4 Hubert Department of Global Health, Rollins School of Public Health, Emory University, Atlanta, GA, USA

## Introduction

Beta-lactam antibiotics are widely used due to their broad spectrum of activity and low toxicity. Carbapenems are beta lactams that can be used to treat many organisms that have intrinsic or acquired resistance to other beta lactam subclasses (eg, cephalosporins). However, with increasing use, resistance to carbapenems has emerged, mostly concerningly due to several specialized beta-lactamase enzymes known as carbapenemases. Carbapenem-resistant Enterobacterales (CRE) are an important subset of carbapenem-resistant organisms since Enterobacterales are part of the normal human microbiome and a frequent cause of bloodstream infections.^1^

Multiplex rapid diagnostic tests are increasingly being used in clinical microbiology laboratories to identify pathogens and genotypic predictors of antimicrobial resistance directly from positive blood culture bottles.^2–4^ These tests offer the potential both to rapidly detect pathogens not covered by empiric antimicrobial therapy as well as to identify patients being treated empirically with broad spectrum therapy who can be safely changed to narrower spectrum antibiotic therapy. A key component to incorporating these rapid diagnostic test results into patient care in the context of emerging carbapenem resistance is to understand the proportion of carbapenem-resistant isolates with a carbapenemase gene detectable by the currently available multiplex panels (ie, sensitivity).

## Methods

We used data from the CDC-funded Georgia Emerging Infections Program (EIP) surveillance of CRE cases to better understand the reliability of rapid blood culture diagnostics for detecting carbapenem resistance. CRE isolates were collected by the Georgia EIP as part of ongoing population-based surveillance from July 2011 to December 2018. The EIP case definition required residence in an 8-county metropolitan area around Atlanta, GA and collection from a normally sterile site or urine. From 2011 to 2015, cases must be resistant to at least one carbapenem (minimum inhibitory concentration [MIC] >= 4), excluding ertapenem, and resistance to all third generation cephalosporins tested (ceftriaxone and cefotaxime MIC >= 4 and ceftazidime MIC >= 16). From 2016 to 2018, the case definition was changed to include ertapenem (MIC >= 2) and removed the requirement for cephalosporin resistance. A convenience sample of up to 120 CRE isolates per year were selected to be sent to CDC for further characterization. Broth microdilution antimicrobial susceptibility testing (AST) was performed as previously described^5^ on all viable isolates to confirm the phenotypic case definition. A subset of confirmed isolates were selected for whole genome sequencing (WGS) performed using the Illumina MiSeq or NovaSeq system. Confirmatory broth microdilution and WGS were performed asynchronously, and the results were not provided to the referring clinical sites.

We analyzed isolates with annotated WGS data for the presence of carbapenemases^5^ (ie, IMP, KPC, NDM, OXA-23 and—48, VIM) that are predicted to be detectable by rapid blood culture diagnostics currently available in the USA.^2–4^ Single and paired proportion z-tests were calculated in RStudio.^6^

## Results

During the study period, 295 CRE isolates were identified and had broth microdilution AST and WGS completed (Table 1). Of these, 276 were phenotypically resistant to ertapenem and 214 were phenotypically resistant to meropenem upon confirmatory testing by broth microdilution. No isolate was susceptible to ertapenem but resistant to meropenem. For confirmed ertapenem resistant isolates, 223/276 (80.8%, 95% CI: 75.5–85.2%) had a carbapenemase predicted to be detectable by commercially available rapid diagnostic tests. For confirmed meropenem resistant isolates, 199/214 (93.0%, 95% CI: 88.5–95.9%) had a carbapenemase predicted to be detectable by commercially available rapid diagnostic tests. Ertapenem monoresistant isolates were less likely to have a detected carbapenemase compared to isolates with meropenem resistance (38.7% vs 93.0%, *P* < .0001).

**Table 1.** Descriptive statistics of 295 CRE isolates referred for confirmatory broth microdilution antimicrobial susceptibility testing

			Carbapenemases^a^				
			ANY		KPC	NDM	OXA
Organisms	N	%	N	%	N	N	N
*Enterobacter cloacae*	33	11.2	14	42.4	13	1	–
*Escherichia coli*	41	13.9	22	53.7	20	2	–
*Klebsiella aerogenes*	8	2.7	1	12.5	1	–	–
*Klebsiella oxytoca*	4	1.4	2	50.0	2	–	–
*Klebsiella pneumoniae*	207	70.2	182	87.9	177	4	3
*Klebsiella variicola*	2	0.7	2	100.0	2	–	–
Source							
Urine	239	81.0	184	77.0	177	6	3
Sterile site (incl. blood)	56	19.0	39	69.6	38	1	–
Ertapenem monoresistance							
Yes	62	22.5	24	38.7	24	–	–
No	214	77.5	199	93.0	191	7	3
Total	276^b^		223	80.8	215	7	3

^a^Presence of carbapenemases genes, including IMP, KPC, NDM, OXA-23 and—48, and VIM detected by whole genome sequencing. No isolates had VIM or IMP carbapenemases. ^b^19 isolates were not resistant to ertapenem or meropenem upon confirmatory broth microdilution testing.

## Discussion

In a multi-year laboratory and population-based active surveillance program of CRE in a large US city, 80.8% of ertapenem resistant and 93.0% of meropenem-resistant isolates had carbapenemase genes predicted to be detectable by commercially available rapid diagnostic tests for blood cultures. These findings suggest that current commercially available panels are highly predictive of meropenem resistance and moderately predictive of ertapenem resistance in the study population.

All isolates in this study were from the Atlanta, GA metropolitan area which may limit the generalizability of the findings. A recent Dutch national epidemiologic study found 849/892 (95.2%) of CRE isolates had a carbapenemase-encoding gene with the vast majority being genes predicted to be detectable by currently available rapid diagnostic tests (eg, KPC, NDM, OXA-48).^7^ These studies surveilled similar populations with respect to a low baseline prevalence of CRE, suggesting that these results may be generalizable in low prevalence populations. Further work is needed in settings with higher prevalence of CRE where estimates of the proportion of CRE attributable to carbapenemases have been more variable.^8,9^

Interestingly, there is emerging evidence that prior carbapenem exposure is a risk factor for non-carbapenemase based carbapenem resistance.^8^ This suggests that currently available panels are at risk for missing isolates with acquired resistance (eg, porin mutations) due to prior carbapenem exposure and underscores the importance of antimicrobial stewardship programs in decreasing unnecessary carbapenem use.^10^ Notably, none of the isolates in our study had a rapid diagnostic test performed and so we are not able to directly compare the performance of rapid diagnostic tests with WGS data.

In conclusion, active surveillance of CRE in a low prevalence setting complemented by WGS reveals that most CRE would be detectable by currently available rapid diagnostic tests for bloodstream infection. Ongoing surveillance is needed to determine if panel sensitivity changes over time as increasing use of carbapenems could result in changing epidemiology of resistance mechanisms and decreased sensitivity of the currently available panels.
